# Efficacy and safety of remimazolam besylate in bronchoscopy for adults: A multicenter, randomized, double-blind, positive-controlled clinical study

**DOI:** 10.3389/fphar.2022.1005367

**Published:** 2022-10-13

**Authors:** Ying-Yong Zhou, Shu-Ting Yang, Kai-Ming Duan, Zhi-Hong Bai, Yun-Fei Feng, Qu-Lian Guo, Zhi-Gang Cheng, Hui Wu, Wang-Ning Shangguan, Xiao-Min Wu, Chun-Hui Wang, Xiao-Qing Chai, Guo-Hai Xu, Cun-Ming Liu, Gao-Feng Zhao, Chun Chen, Bao-An Gao, Li-E Li, Min Zhang, Wen Ouyang, Sai-Ying Wang

**Affiliations:** ^1^ Department of Anesthesiology, The Third Xiangya Hospital of Central South University, Changsha, China; ^2^ Department of Anesthesiology, Xiangya Hospital Central South University, Changsha, China; ^3^ Department of Anesthesiology, The First Affiliated Hospital of Wenzhou Medical University, Wenzhou, China; ^4^ Department of Anesthesiology, The Second Affiliated Hospital and Yuying Children’s Hospital of Wenzhou Medical University, Wenzhou, China; ^5^ Department of Anesthesiology, Zhejiang Provincial People’s Hospital, Hangzhou, China; ^6^ Department of Anesthesiology, The Fourth Affiliated Hospital of Anhui Medical University, Hefei, China; ^7^ Department of Anesthesiology, The First Affiliated Hospital of USTC, Division of Life Sciences and Medicine, University of Science and Technology of China, Hefei, China; ^8^ Department of Anesthesiology, The Second Affiliated Hospital of Nanchang University, Nanchang, China; ^9^ Department of Anesthesiology, Jiangsu Province Hospital, NanJing, China; ^10^ Department of Anesthesiology, Guangdong Provincial Hospital of Traditional Chinese Medicine, Guangzhou, China; ^11^ Department of Anesthesiology, Yichang Central People’s Hospital, Yichang, China; ^12^ Yichang Humanwell Pharmaceutical Co., Ltd, Yichang, China

**Keywords:** remimazolam, bronchoscopy, sedation, hypotension, injection pain

## Abstract

**Background:** With the development of fiberoptic bronchoscopy in the diagnosis and treatment of various pulmonary diseases, the anesthesia/sedation requirements are becoming more demanding, posing great challenges for patient safety while ensuring a smooth examination/surgery process. Remimazolam, a brand-new ultra-short-acting anesthetic, may compensate for the shortcomings of current anesthetic/sedation strategies in bronchoscopy.

**Methods:** This study was a prospective, multicenter, randomized, double-blind, parallel positive controlled phase 3 clinical trial. Subjects were randomized to receive 0.2 mg/kg remimazolam besylate or 2 mg/kg propofol during bronchoscopy to evaluate the efficacy and safety of remimazolam.

**Results:** A total of 154 subjects were successfully sedated in both the remimazolam group and the propofol group, with a success rate of 99.4% (95%CI of the adjusted difference −6.7 × 10%–6% to −5.1 × 10%–6%). The sedative effect of remimazolam was noninferior to that of propofol based on the prespecified noninferiority margin of −5%. Compared with the propofol group, the time of loss of consciousness in the remimazolam group (median 61 vs. 48s, *p* < 0.001), the time from the end of study drug administration to complete awakening (median 17.60 vs. 12.80 min, *p* < 0.001), the time from the end of bronchoscopy to complete awakening (median 11.00 vs. 7.00 min, *p* < 0.001), the time from the end of study drug administration to removal of monitoring (median 19.50 vs. 14.50 min, *p* < 0.001), and the time from the end of bronchoscopy to removal of monitoring (median 12.70 vs. 8.60 min, *p* < 0.001) were slightly longer. The incidence of Adverse Events in the remimazolam group and the propofol group (74.8% vs. 77.4%, *p* = 0.59) was not statistically significant, and none of them had Serious Adverse Events. The incidence of hypotension (13.5% vs. 29.7%, *p* < 0.001), hypotension requiring treatment (1.9% vs. 7.7%, *p* = 0.017), and injection pain (0.6% vs. 16.8%, *p* < 0.001) were significantly lower in the remimazolam group than in the propofol group.

**Conclusion:** Moderate sedation with 0.2 mg/kg remimazolam besylate is effective and safe during bronchoscopy. The incidence of hypotension and injection pain was less than with propofol, but the time to loss of consciousness and recovery were slightly longer.

**Clinical Trial Registration:** clinicaltrials.gov, ChiCTR2000039753

## Introduction

Bronchoscopy plays an important role in the diagnosis and treatment of lung diseases. Since the first invention of flexible bronchoscopy in 1968 (Ikeda et al., 1968), the number and complexity of interventional bronchoscopy procedures have increased significantly. Sedation was initially rarely used in bronchoscopy, but was difficult to perform clinically due to patients’ anxiety, restlessness, pain, cough, and dyspnea (Poi et al., 1998; Aljohaney, 2019), thus compromising the therapeutic effect and leaving patients with an unpleasant experience. The British Thoracic Society guidelines for flexible bronchoscopy in adults recommend moderate sedation without contraindication (Rand et al., 2013), which can make patients more comfortable and reduce anxiety, cough, and dyspnea. At the same time, complications such as cardiac arrhythmias and hypertension can be reduced (Matot and Kramer, 2000; Gonzalez et al., 2003). Bronchoscopy insertion and operation typically result in irritation similar to surgical incisions, and because of the susceptibility of the common airway to hypoxemia, providing adequate depth of sedation to ensure a smooth examination/procedure also presents a significant patient safety challenge (Matot and Kramer, 2000; Rand et al., 2013; Goudra et al., 2015). Both the guidelines (Rand et al., 2013) and expert consensus (Wahidi et al., 2011) recommend sedation in patients undergoing bronchoscopy without contraindications. However, under procedural sedation, although it improves patient tolerability, respiratory depression and hypoxemia are still high (Stolz et al., 2004; Schlatter et al., 2011). It can even lead to serious AE such as cardiac arrhythmias and coronary dysfunction (Shrader and Lakshminarayan, 1978). The use of an LMA during fiberoptic bronchoscopy can improve hypoxemia, provide airway support, and allow comfortable access to the glottis, especially in patients with subglottic obstruction and high-risk patients (Biro et al., 2001; Abdelmalak et al., 2012; Alon et al., 2017).

Managing sedation levels according to pharmacologic principles and selecting appropriate sedatives are key to the safe management of anesthesia during bronchoscopy (Goudra et al., 2015). Propofol and midazolam (Rand et al., 2013) are the most commonly used intravenous sedatives for bronchoscopy in clinical practice. Both are widely used in the clinical setting as common medications, but they also have their limitations. Infusion of propofol often causes hypotension and pain at the injection site; other adverse reactions such as hypersensitivity, bronchospasm, thrombophlebitis, dyslipidemia, and bacterial infections may even occur (Marik, 2004; Sim et al., 2009; Desousa, 2016; Dinis-Oliveira,2018). Midazolam has a slow onset of action (3–5 min) and a long half-life (approximately 1.5–2.5 h) (Nordt and Clark, 1997; Olkkola and Ahonen, 2008), so titration over several minutes is required to take effect, while rapid titration may lead to accidental overdose. In addition, the metabolites α-1-hydroxymidazolam and 4-hydroxymidazolam also have a drug effect, which can easily lead to delayed awakening and is not conducive to short-term outpatient procedures such as bronchoscopies. Therefore, the development of a new intravenous sedative that can compensate for the above shortcomings remains the most important clinical need.

Remimazolam besylate for injection (Kilpatrick et al., 2007; Rogers and McDowell, 2010) is a new type of benzodiazepine that is one of the ultra-short-acting sedative/anesthetics. It acts by enhancing the activity of GABAA receptors with γ-subunits. It acts on central GABAa receptors, opening channels, increasing chloride influx, and causing hyperpolarization of nerve membranes to inhibit neuronal activity. Remimazolam besylate has the advantages of rapid onset of action, good aqueous solubility, and a short elimination half-life (approximately 0.75 h). It is rapidly hydrolyzed by tissue carboxylesterase into inactive carboxylic acid metabolites and is not dependent on P450 enzyme metabolism in cells. It also has a specific antagonist (flumazenil) (Schüttler et al., 2020) (Sheng et al., 2020). It has been developed for sedation during therapeutic and diagnostic procedures, induction and maintenance of general anesthesia, and sedation in the intensive care unit and is expected to show a benefit for physicians and patients during bronchoscopic diagnosis and treatment. Although the use of LMA and remimazolam may provide advantages for the development of bronchoscopy, there is no clinical study on the use of remimazolam for bronchoscopy under LMA. The aim of this study was to evaluate the efficacy and safety of remimazolam besylate during bronchoscopy. We hypothesized that remimazolam is non-inferior to propofol with respect to efficacy of sedation.

## Materials and methods

### Study design and procedures

According to the Declaration of Helsinki, this multicenter, randomized, double-blind, parallel, active-controlled phase 3 clinical trial was conducted in 11 medical centers in China to evaluate the efficacy and security of remimazolam besylate for injection in bronchoscopy. The study was approved by the ethics committee of each participating hospital. The trial was prospectively registered at www.chictr.org.cn (ChiCTR2000039753). Written informed consent was obtained from all patients before enrollment.

The trial plans to recruit 310 eligible patients and randomly divide them into two groups in a 1:1 ratio. The experimental group was the remimazolam besylate injection group, and the control group was the propofol medium/long-chain fat emulsion (propofol-MCT/LCT) injection group. The duration of the trial included the screening period (within 7 days before bronchoscopy) and the treatment period (on the day of bronchoscopy). Follow-up period (within 2–4 days after bronchoscopy).

Inclusion criteria: Age 18–75 years, male or female; American Society of Anesthesiologists (ASA) Class I-III, Body Mass Index (BMI) 18–30 kg/m2, subjects planning to undergo bronchoscopy for diagnosis and/or treatment under laryngeal mask ventilation, Breathing rate 10 to 24 times/min, blood oxygen saturation ≥93% when breathing air, systolic blood pressure≥ 90 mmHg, diastolic blood pressure ≥55 mmHg, and resting heart rate 50 to 100 beats per minute. Ability to understand and voluntarily sign the informed consent form and willing to complete the study according to the requirements of the study protocol. Exclusion Criteria: Patients with contraindications to deep sedation/general anesthesia or a history of sedation/anesthesia accidents; Allergic or contraindicated to benzodiazepines, opioids, propofol, flumazenil, naloxone, lidocaine, and other drugs and their components; Screening/psychiatric history; history of cardiovascular, respiratory, and digestive systems affecting the study; abnormal and clinically significant laboratory tests during the screening period; Modified Mallampati Score with grade III and above. For detailed criteria, see the Chinese Clinical Trials Registry website (https://clinicaltrials.gov/; ChiCTR2000039753).

This study used a group randomization, stratified by center and a double-blind design. Because the two drugs can be easily distinguished externally, this study used an evaluator (blind) and an administration researcher (nonblind) to ensure double-blind implementation. The process of randomization, drug dispensing, and drug administration was performed by the investigator administering the drugs. The investigator for the evaluation gave instructions, and the investigator for drug administration, who was not blind, gave the appropriate drugs according to the instructions.

All eligible subjects began aerosol inhalation of 10 ml of 2% lidocaine (recommended duration not less than 15 min) within 60 min before drug administration for local airway anesthesia. Oxygen was administered at a flow rate >6 L/min for at least 3 min before study drug administration. A dose of 2 μg/kg fentanyl citrate was administered intravenously in 15 s (±5s), and the study drug was administered intravenously 3 min (±5 s) after fentanyl administration. According to previous studies and literature reports (Sheng et al., 2020), the recommended induction dose of remimazolam during general anesthesia in Chinese subjects is 0.2 mg/kg. The stimulation intensity during bronchoscope insertion is the same as during induction of general anesthesia. Therefore, remimazolam besylate (Yichang Renfu, lot number: 90T0703) and propofol-MCT/LCT (Fresenius Kabi Austria GmbH, lot number: 16NK5658) were administered at initial doses of 0.2 mg/kg and 2 mg/kg within 1 min, respectively. The level of sedation was assessed using the Modified Observer’s Assessment of Alertness/Sedation Scale (MOAA/S, Chernik et al., 1990). Sedation scores were defined as follows: 5 = responds readily to name spoken in normal tone; 4 = lethargic response to name spoken in normal tone; 3 = responds only after name is called loudly and/or repeatedly; 2 = responds only after mild prodding or shaking; 1 = responds only after painful trapezius squeeze, 0 = does not respond to painful trapezius squeeze. If the subject did not reach loss of consciousness (LOC, MOAA/S ≤ 1) within 2 min after the initial dose, additional doses of 0.1 mg/kg remimazolam besylate or 0.75 mg/kg propofol-MCT/LCT could be administered within 20 ± 5s. The interval between additional doses was ≥1 min until the MOAA/S score was ≤1.The maximum number of additional administrations was three times in the sedation induction phase. If the MOAA/S score was still >1 at 1 min after the three additional administrations in the induction phase, this was recorded as sedation failure. Subjects who fail sedation could only use propofol-MCT/LCT as rescue sedation to complete the diagnostic and treatment process. When subjects completed the induction phase and reached the standard of LMA placement (MOAA/S ≤ 1), the appropriate LMA type was placed and oxygen was administered, and bronchoscopy diagnosis and treatment began. 2% lidocaine was injected at 2–5 ml when the bronchoscopy passed the glottis or carina. During intervention , remimazolam besylate at a dosage of 0.1 mg/kg/time or propofol-MCT/LCT at a dosage of 0.75 mg/kg/time was administered to maintain the sedation level according to the subjects’ condition (body movement, swallowing, eye opening, coughing, and other symptoms of inadequate anesthesia). The time of additional administration was 20 s (±5 s). The interval of additional administration should be ≥ 2 min. During bronchoscopy, fentanyl was added according to the subject’s condition. The additional dose of fentanyl was 25 μg, and the total additional dose did not exceed 200 μg.

Vital signs were collected once during the screening phase and the follow-up phase. During the treatment period, vital signs were recorded every 2 min (±30 s) after the start of study drug administration until subjects reached the criteria for removal of monitoring before discharge from the recovery room (third consecutive Aldrete score ≥9). The MOAA/S score was determined once before the injection of fentanyl citrate and immediately (0 min) at the start of study drug administration. The MOAA/S score was continuously evaluated after the start of study drug administration until the subjects reached LOC, and the LOC time was recorded in seconds. The MOAA/S score was evaluated immediately after bronchoscopy and then evaluated and recorded every 1 min until the subject completed awakening (the third consecutive MOAA/S score = 5). The Aldrete score was then evaluated every 2 min until the subject reached the removal of monitoring. At the end of bronchoscopy, the subjects’ wakefulness, releasing from monitoring and administration site were scored according to protocol requirements. The LMA could be removed after bronchoscopy when the subject was fully awake.

## Efficacy assessment

### Primary efficacy outcomes

The primary efficacy outcome of the study was the success rate of sedation, which had to meet the following two conditions: 1) Completed bronchoscopy diagnosis and treatment; 2) No useed of sedatives during the induction phase.

### Secondary efficacy outcomes

(1) The time from the start of study drug administration to the first MOAA/S ≤ 1; 2) The time from the end of study drug administration to complete awakening (the first time that the three consecutive MOAA/S scores were 5 ); 3) The time from the end of bronchoscopy (immediately after the bronchoscopy probe leaves the mouth) to full awakening; 4) The time from the end of study drug administration to the removal of monitoring (the first time that three consecutive Aldrete scores≥9 were achieved); 5) The time from the end of bronchoscopy (immediately after the bronchoscope probe left the mouth) to the removal of monitoring; (6) The proportion of subjects who had used a rescue medication.

## Safety assessment

Safety Assessment included Adverse Events (AEs) injection pain, vital signs, physical examination, clinical laboratory tests (blood routine, blood biochemistry, urine routine, coagulation function), electrocardiogram, and premature termination of study treatment due to AEs. All AEs were characterized by type, severity, severity, and association with treatment according to the Common Terminology Criteria for AEs 5.0 (CTCAEs 5.0). Depending on whether there was a reasonable time sequence between the occurrence of AE and the study drug, what type of drug reaction occurred, and whether the reaction was relieved, resolved, or recurred after discontinuation of the drug, investigators rated the association between AE and the study drug as unrelated, unlikely or distantly related, possibly related, probably related, definitely related. Every adverse event was actively treated, regardless of whether it was causally related to the study drug. Serious Adverse Events (SAEs) were identified when daily functions were impaired or life-threatening and hospitalization or prolonged hospitalization was required. Sedation-related AEs, including respiratory depression, hypotension, and hypotension requiring treatment, were assessed from study drug administration until patient discharge. Respiratory depression was defined as pulse oxygen saturation (SpO2) < 90%; hypotension was defined as systolic blood pressure (SBP) < 90 mmHg or 20% lower than baseline; treatment-emergent hypotension was defined as systolic blood pressure (SBP) ≤90 mmHg or lower 30% reduction in baseline.

## Statistical analysis

All statistical analyzes were performed using SAS ver. 9.4 (SAS Institute Inc., Cary, NC, United States). According to the phase II induction and maintenance of general anesthesia study, the sedation success rate in both the control and remimazolam groups in this study was expected to be 98%, and the noninferiority margin was -5%. One-tailed test was used, *p* value was 0.025, and powe value was 0.8. The remimazolam group and the control group were in a 1:1 ratio, and the software PASS 14.0 was used to estimate the sample size. There were 124 patients in the remimazolam group and 124 patients in the control group. Considering the dropout rate of about 20%, a total of 310 patients should be enrolled in this study, including 155 in the remimazolam group and 155 in the control group.

For the proportion of successful sedation as the primary efficacy outcome, the differences between groups were compared using Chisq’s test or Fisher’s exact test, and the 95% confidence interval for the difference was calculated using Newcombe’s method. Using center and sex as stratification factors, a logistic regression model was used to calculate the corrected rate difference. The bootstrap method was used to run 1,000 samples to estimate the standard error of the corrected rate difference, and then the two-sided 95% confidence interval of the corrected rate difference was calculated. The noninferiority margin of −5% was used to assess whether the experimental group was noninferior to the control group. The rank sum test (Wilcoxon) or the Cochran-Mantel-Haenszel (CMH) test was used for the measurement data of the secondary efficacy outcomes, and the Chisq test or Fisher’s exact test was used for the count data to compare the differences between groups.

In the safety analyzes, the incidence of AEs and drug-related AEs between the two groups was tested using the chi-square test, Fisher’s exact test, or *t* test. Continuous variables were expressed as mean ± standard deviation (SD) or median with range (min, max), whereas categorical variables were expressed as percentage.

Data management and statistical analysis were performed by the third party Shanghai Bojia Pharmaceutical Technology Co., Ltd. All statistical tests were two-sided, and the *p* value <0.05 was considered statistically significant.

## Results

### Disposition and baseline characteristics of subjects

**FIGURE 1:**
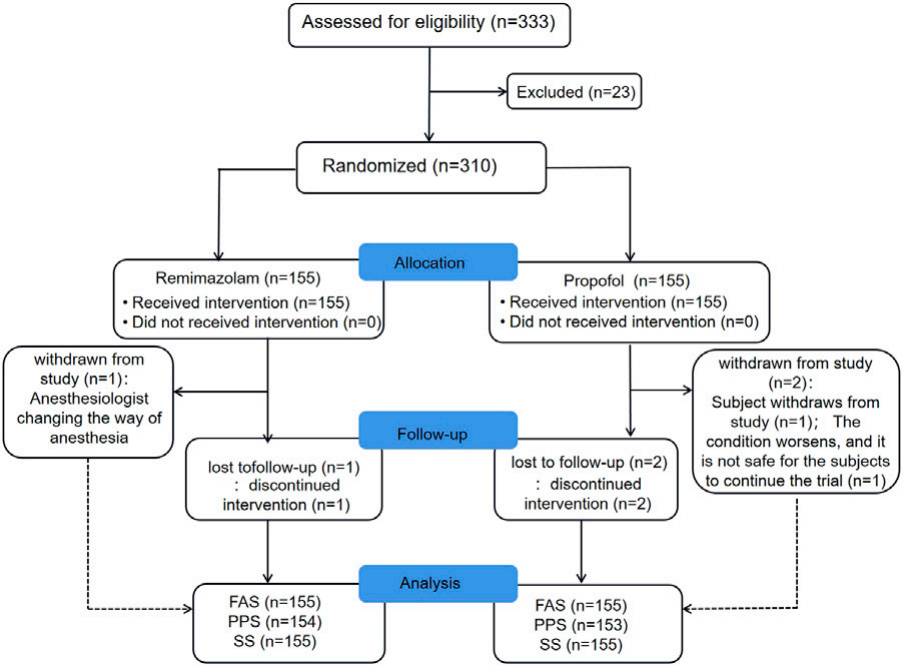
Enrollment flow diagram.FAS full analysis set, PPS per-protocol set, SS safety set.

**FIGURE 2:**
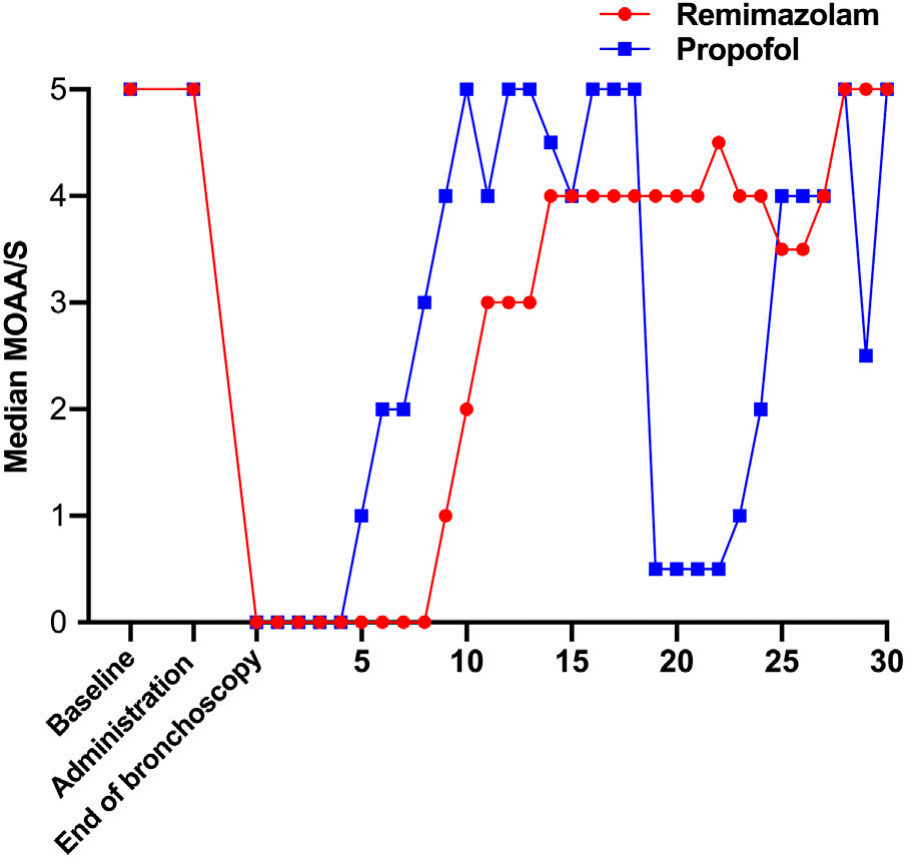
The time course of the MOAA/S score for both groups.

From November 2020 to June 2021, a total of 333 subjects from 11 centers were screened, of whom 23 failed screening and 310 were randomly enrolled into the study. The experimental group (remimazolam besylate for injection) and control group (propofol-MCT/LCT) were 155 cases and 155 cases, respectively, and 307 cases were completed. There were 154 cases in the test group and 153 cases in the control group. 3 Subjects dropped out of the study prematurely, 1 case in the experimental group and 2 in the control group. Finally, the data of 310 subjects in FAS and SS, and the data of 307 subjects in PPS, and the study schedule (Figure 1) were analyzed. The demographic characteristics, vital signs, and electrocardiogram of the two groups were comparable (Table 1). There were no significant differences in the duration and types of bronchoscopy, the dosage of fentanyl before and during the study drug administration between the two groups (Table 2). The time course of MOAA/S scores from induction to full recovery in the two groups is shown in Figure 2.

**TABLE 1:** Demographics, vital signs, and ECG baseline characteristics (FAS).

Characteristics	Remimazolam (n = 155)	Propofol (n = 155)	*p*-value
Age (years)	49.7 ± 13.4	51.9 ± 11.8	0.13
Height (cm)	162 ± 8.29	162 ± 8.03	0.96
Weight (kg)	60.8 ± 9.57	62.0 ± 9.71	0.25
BMI(kg/m2)	23.0 ± 2.62	23.5 ± 2.75	0.12
Gender			
Female	83 (53.5)	73 (47.1)	0.20
Race			
Han	145 (93.5)	142 (91.6)	0.52
Other	10 (6.5)	13 (8.4)	
History of drug allergy	12 (7.7)	9 (5.8)	0.50
ASA status			
I	27 (17.4)	19 (12.3)	0.28
II	126 (81.3)	135 (87.1)	
III	2 (1.3)	1 (0.6)	
Modified mallampati score			
I	55 (35.5)	50 (32.3)	0.55
II	100 (64.5)	105 (67.7)	
General health			
Normal	136 (87.7)	133 (85.8)	0.60
NCS	2 (1.3)	2 (1.3)	
CS	17 (11.0)	20 (12.9)	
Chest imaging examination			
Normal	3 (1.9)	4 (2.6)	0.82
NCS	39 (25.2)	35 (22.6)	
CS	113 (72.9)	116 (74.8)	
HR (bpm)	73.7 ± 11.39	74.2 ± 11.73	0.69
RR (bpm)	16.4 ± 3.28	16.0 ± 3.08	0.20
SBP (mmhg)	127 ± 16.76	129 ± 16.66	0.45
DBP(mmhg)	77.9 ± 10.28	80.1 ± 9.80	0.053
SPO2 (%)	98.6 ± 1.59	98.3 ± 1.60	0.070
T (°c)	35.9 ± 0.75	35.9 ± 0.75	0.97
ECG			
PR interval (ms)	147 ± 21.88	151 ± 20.06	0.054
QRS interval (ms)	90.8 ± 10.97	89.7 ± 10.68	0.38
QT interval (ms)	388 ± 26.44	382.±26.33	0.057
QTc interval (ms)	419 ± 21.45	414 ± 23.28	0.068
ECG clinical significance			
Normal	96 (61.9)	101 (65.2)	0.75
NCS	55 (35.5)	49 (31.6)	
CS	4 (2.6)	5 (3.2)	

Data are presented as mean ± SD, or n (%); BMI, body mass index; SBP, systolic blood pressure; MAP, mean arterial pressure; DBP, diastolic blood pressure; ASA, American Society of Anesthesiologists; NCS, Not Clinically Significant; CS, Clinical Significant.

**TABLE 2:** Analysis of fentanyl dosage, duration and types of bronchoscopy (FAS).

Index	Remimazolam (n = 155)	Propofol (n = 155)	*p*-value
Duration of bronchoscopy (min)	13.8 ± 14.2	12.6 ± 12.3	0.44
Types of bronchoscopy			
Check the airways	66 (42.6)	66 (42.6)	0.22
Biopsy	66 (42.6)	75 (48.4)	
EBUS-TBNA	7 (4.5)	8 (5.2)	
Bronchoalveolar lavage	15 (9.7)	6 (3.9)	
Bronchial foreign body removal	1 (0.6)	0 (0)	
Fentanyl dosage before study drug administration (μg)	121 ± 19.72	124 ± 19.50	0.24
Fentanyl dosage during study drug administration (μg)	36.4 ± 16.43	32.4 ± 11.63	0.29

Data are presented as mean ± SD or n (%); EBUS-TBNA,endobronchial ultrasound-guided transbronchial needle aspiration.

### Efficacy assessment

#### Primary efficacy outcomes

In FAS or PPS, no statistically significant difference was found between the two groups on the primary efficacy outcome (*p* > 0.999). After adjustment for relevant factors at the study center, the difference in performance between the two groups was greater than the prespecified noninferiority value (−5%) (Table 3). According to the prespecified noninferiority threshold (−5%), the proportion of successful sedation in the remimazolam group was noninferior to that of the propofol group during bronchoscopy. In both groups, sedatives were not used during the induction phase. In the remimazolam group, 144 subjects (92.9%) achieved the depth of anesthesia required for LMA placement without additional medication during induction, 8 subjects (5.2%) received 0.1 mg/kg once to achieve the appropriate depth of sedation, and 3 subjects (1.9%) received 0.1 mg/kg three times to achieve the appropriate depth of sedation. No additional medications were used in the propofol group. Compared with the propofol group, the number of patients who needed additional doses for induction was higher for remimazolam (11/155 vs. 0/155, *p* < 0.001).

**TABLE 3:** Proportional analysis of sedation success (FAS/PPS).

Set	Group	Success	*p*-value	Difference (%)	95% CI(Wald)
FAS	Remimazolam	154 (99.4%)	>0.999	0	−3.0 to 3.0
	Propofol	154 (99.4%)		−5.9 × 10–6^a^	−6.7 × 10–6 to −5.1 × 10–6^a^
PPS	Remimazolam	154 (100%)	NA	0	−2.48 to 2.45
	Propofol	153 (100%)		b	

FAS, full analysis set; PPS, per-protocol set. A After adjusting the research centers factors. B The power of both groups was 100%, and no logistic regression model correction analysis was performed.

#### Secondary efficacy outcomes

Compared with propofol, the median time from the onset of study drug administration to the first MOAA/S score ≤1 was slightly longer in the remimazolam group (61 vs. 48s, *p* < 0.001) (Table 4). The results showed that the induction time of 2 mg/kg propofol was slightly shorter than that of 0.2 mg/kg remimazolam, and an adequate level of sedation was achieved within the clinically acceptable time in both groups.

**TABLE 4:** Secondary efficacy outcomes (FAS).

Secondary end-points	Remimazolam (n = 155)	Propofol (n = 155)	*p*-value
Time from the start of drug administration to the first MOAA/S score ≤1 (s)	61.0 (49.0–85.0)	48.0 (40.0–70.0)	<0.001
Time from the end of drug administration to complete awakening (min)	17.60 (13.30–23.50)	12.80 (9.90–16.20)	<0.001
Time from the end of bronchoscopy to complete awakening (min)	11.00 (7.00–16.10)	7.00 (4.00–10.00)	<0.001
Time from the end of drug administration to the removal of monitoring (min)	19.50 (15.20–25.30)	14.50 (11.70–17.80)	<0.001
Time from the end of bronchoscopy to the removal of monitoring (min)	12.70 (9.00–17.70)	8.60 (5.50–12.30)	<0.001

Data are presented as median and range.

Compared with propofol, the median time from the end of drug administration to complete awakening, The median time from the end of bronchoscopy to full awakening, The median time from the end of drug administration to the removal of monitoring, and the median time from the end of bronchoscopy to the removal of monitoring were slightly longer (Table 4). Anesthesia recovery time and total recovery time of subjects in the propofol group were significantly shorter than those in the remimazolam group, but both were within the clinically acceptable time.

### Safety Assessment

A total of 310 subjects were enrolled in this trial, and all subjects in the experimental group (*n* = 155) and the control group (*n* = 155) were included in the safety set (SS) analysis set.

The SS set data showed that the incidence of AEs after study drug administration was 116 cases (74.8%) in the experimental group and 120 cases (77.4%) in the control group, and there was no significant difference between the groups (*p* = 0.59). The incidence of adverse reactions was 92 cases (59.4%) in the experimental group and 103 cases (66.5%) in the control group, and there was no significant difference between the groups (*p* = 0.20). In the control group, there was 1 case (0.6%) case in the control group who had AEs that led to withdrawal from the study and was considered an adverse reaction. In the experimental group, there was no Adverse Event leading to withdrawal in the experimental group, and there was no significant difference between the groups (*p* > 0.999). By the CTCAE 5.0 standard, the severity of AEs did not exceed grade 3 in either group, and the severity of Adverse Events was 1–2 in both groups, except for 1 case (0.6%) in the experimental group in which a grade 3 AE occurred (which was scored as an adverse reaction). There was no significant difference between the two groups in the incidence of AEs and adverse reactions that lead to dose escalation of study drug and in the incidence of AEs and adverse reactions that led to action. The incidence of adverse reactions related to laboratory test indicators did not exceed 5% in either group during the follow-up period, the severity did not exceed grade 2, and no SUEs/serious reactions occurred. No SAEs or AEs leading to permanent drug withdrawal or death occurred in either group during the entire study period (Table 5).

**TABLE 5:** Analysis of the incidence of AEs and adverse reactions (SS).

Index	Remimazolam (*n* = 155)	Propofol (*n* = 155)	*p*-value
AEs	116 (74.8)	120 (77.4)	0.59
ADRs	92 (59.4)	103 (66.5)	0.20
SAEs	0 (0)	0 (0)	NA
SADRs	0 (0)	0 (0)	NA
AEs that lead to withdrawal	0 (0)	1 (0.6)	>0.999
ADRs that lead to withdrawal	0 (0)	1 (0.6)	>0.999
AEs that lead to dose escalation	63 (40.6)	55 (35.5)	0.35
ADRs that lead to dose escalation	44 (28.4)	39 (25.2)	0.52
Levels 3–5 AEs	1 (0.6)	0 (0)	>0.999
Levels 3–5 ADRs	1 (0.6)	0 (0)	>0.999
AEs that lead to death	0 (0)	0 (0)	NA
ADRs that lead to death	0 (0)	0 (0)	NA
AEs that lead to action	35 (22.6)	37 (23.9)	0.79
ADRs that lead to action	20 (12.9)	21 (13.5)	0.87

Data are presented as n (%); AEs, Adverse Events; ADRs, Adverse Drug Reaction; SAEs, Serious Adverse Events; SADRs, Serious ADRs.

**FIGURE 3:**
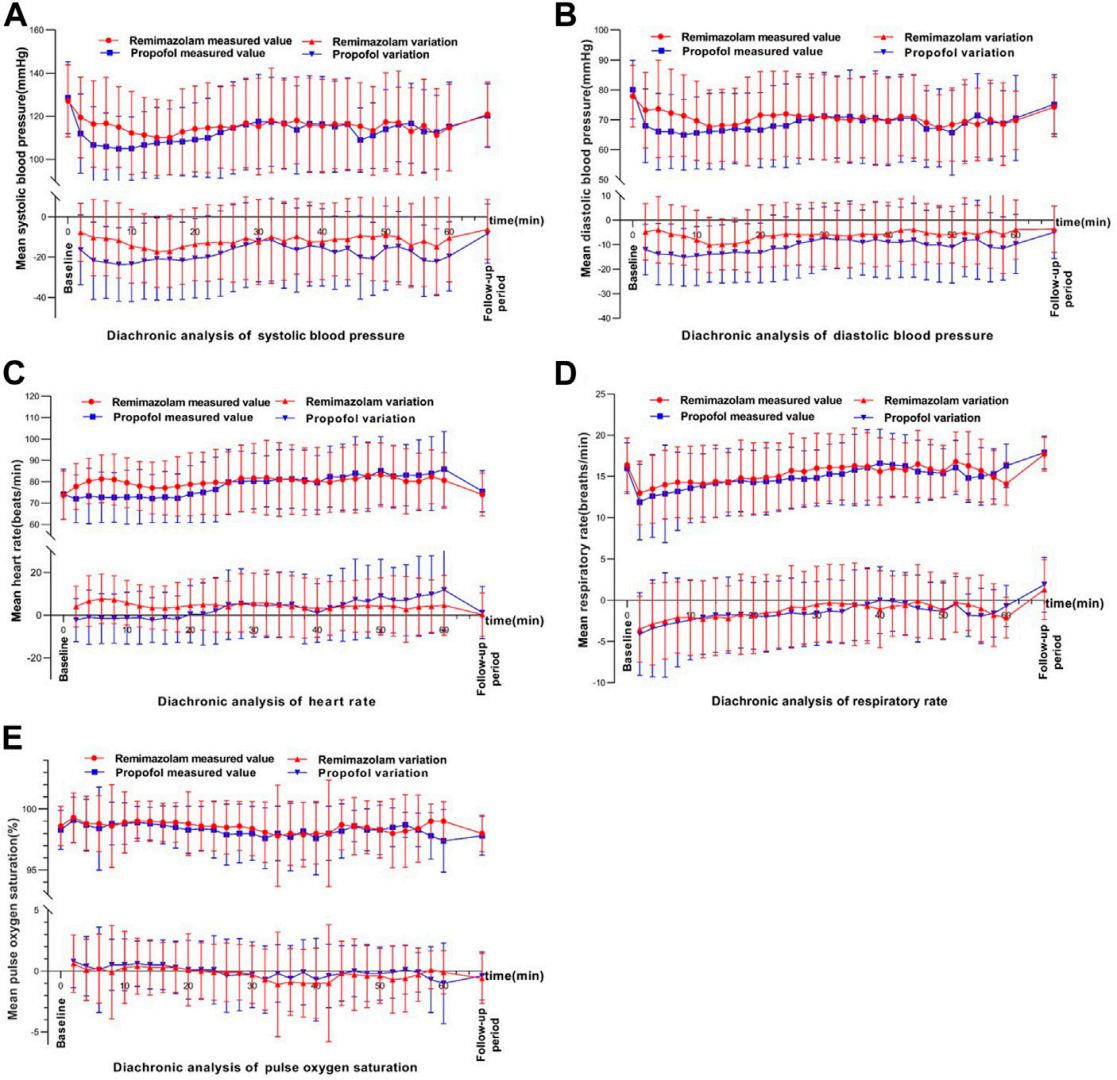
Changes in vital signs over time.In each figure, the upper part shows the diachronic analysis of measured values, and the lower part shows the diachronic analysis of the absoluate change relative to the baseline. **(A)** systolic blood pressure (DBP), **(B)** Diastolic blood pressure, **(C)** Heart rate (HR), **(D)** Respiratory rate and **(E)** Pulse oxygen saturation (SPO2).

The main AEs in the remimazolam group were hypotension, increased blood pressure, vomiting, respiratory depression, decreased respiratory rate, and injection pain. The main AEs in the propofol group were hypotension, injection pain, decreased respiratory rate, respiratory depression, vomiting, increased blood pressure (Table 6). Vital signs during bronchoscopy were shown in Figure 3. Within 10 min of study drug administration, blood pressure in both experimental and control groups showed a downward trend with a decreasing range of 13.61% and 18.98%, respectively. Blood pressure in the remimazolam group decreased less than that in the propofol group. About 30 min after administration, blood pressure values tended to be constant in the two groups and gradually returned to baseline. During sedation for bronchoscopy, the incidence of hypotension, hypotension requiring treatment, and injection pain in the remimazolam group were significantly lower than those in the propofol group (Table 6). Within the first 10 min, there was an increase in heart rate in the remimazolam group (but not in the propofol group) (Figure 3C), and the maximum percentage increase of heart rate was significantly higher than in the propofol group (Table 6). SpO2 was relatively stable in both groups (Figure 3E). The respiratory rate of the subjects in the two groups mainly showed a downward trend (Figure 3D). After LMA placement, respiratory rate remained essentially at baseline in both groups. After completion of bronchoscopy, administration of the study drug was stopped, the LMA was removed, and subjects resumed spontaneous breathing.

**TABLE 6:** Analysis of the incidence of main AEs during sedation (SS).

Index	Remimazolam (*n* = 155)	Propofol (*n* = 155)	*p*-value
Hypotension	22 (14.2)	49 (31.6)	<0.001
Hypotension requiring treatment	3 (1.9)	12 (7.7)	0.017
hypertension	13 (8.4)	5 (3.2)	0.087
vomiting	12 (7.7)	6 (3.9)	0.15
Respiratory depression	9 (5.8)	8 (5.2)	0.80
Reduced respiratory rate	8 (5.2)	11 (7.1)	0.48
Injection pain	1 (0.6)	26 (16.8)	<0.001
Maximum percentage increase of heart rate (the first 10 min)	15.1 ± 17.8	5.3 ± 17.0	<0.001

Data are presented as mean ± SD, or n (%).

## Discussion

This clinical trial was a multicenter, randomized, double-blind, parallel, positive control study with propofol-MCT/LCT to evaluate the efficacy and safety of remimazolam besylate for injection during bronchoscopy. The major findings were: 1) remimazolam besylate can achieve the depth of sedation required for bronchoscopy (without rescue sedation) and its effect is noninferior to propofol; 2) compared with propofol-MCT/LCT, the sedation induction time and recovery time of remimazolam besylate are slightly longer; 3) the drug is safe and superior to propofol-MCT/LCT in terms of injection pain and influence on the circulatory system.

As an ultra-short-acting sedative/anesthetic drug, remimazolam is characterized by rapid onset, good water solubility, rapid clearance, inactive metabolites and specific antagonists (Wiltshire et al., 2012; Schüttler et al., 2020; Sheng et al., 2020), which may be an ideal anesthetic/sedative for bronchoscopy.However, clinical evidence supporting the use of remimazolam for fiberoptic bronchoscopy under LMA is lacking. A multicenter, randomized, double-blind trial by Pastis et al. (2019) demonstrated that remimazolam is effective and safe for moderate sedation during bronchoscopy and has a faster onset of action and shorter neuropsychiatric recovery time than midazolam. Because moderate sedation requires complete dependence on spontaneous breathing to provide oxygenation, the depth of anesthesia and dosage of sedative are less than for general anesthesia under LMA. Therefore, more direct clinical evidence is needed for a medication guide. This study shows that 0.2 mg/kg remimazolam can achieve sufficient depth of anesthesia for LMA implantation in 92.9% of subjects with high confidence. Due to the lack of sequential administration, we cannot confirm that 0.2 mg/kg Remimazolam is the best dose for bronchoscopy under LMA, but it also provides some guidance for clinical administration.

Our study demonstrated that remimazolam besylate for injection and propofol-MCT/LCT can effectively complete bronchoscopy, and both can enable subjects to achieve the depth of sedation required for bronchoscopy. The ratio of successful sedation with remimazolam was not inferior to propofol after correction. However, the LOC time, time to complete awakening, and time to release from monitoring were slightly longer in the remimazolam group. A multicenter, single-blind, randomized, parallel-group phase IIb/III trial by Doi et al. (2020) also showed that two induced doses of remimazolam (6 and 12 mg/kg/h) were noninferior to propofol (2.0–2.5 mg/kg) for general anesthesia. In addition, the incidence of hypotension was lower in the remimazolam group, allowing more time for LOC and extubation. Another phase III clinical trial in colonoscopy also showed that the sedative effect of remimazolam was noninferior to that of propofol, and the time for sedation induction was relatively longer, but recovery was similar (Chen et al., 2020).

Regarding the onset of action of remimazolam, the studies by Doi et al. (2020) and Chen et al. (2020) are consistent with our research findings. Compared with propofol, the longer sedation induction time of remimazolam may be due to its slower equilibration between plasma and effect-site concentration when compared with propofol. For the EEG-effect (assessed by the bispectral index BIS) the effect site equilibration rate ke0 for remimazolam was 0.14 min–1 (Zhou et al., 2020), corresponding to an equilibration half-life T1/2ke0 of 4.9 min. For propofol, ke0 values between 0.26 and 0.57 min-1 have been reported (Struys et al., 2007), corresponding to an equilibration half-life of 1.2–2.7 min. Remimazolam is rapidly eliminated and hydrolyzed by carboxylesterase to an inactive carboxylic acid metabolite, regardless of age and disease status (Wiltshire et al., 2012; Schüttler et al., 2020; Sheng et al., 2020). Chen et al. (2020) showed that the recovery time of remimazolam was similar to that of propofol. Both our results and those of Doi et al. (2020) suggest that the recovery time of remimazolam is slightly longer. The longer time for onset and recovery after remimazolam might also be caused by a slower equilibration between plasma and effect-site concentration when compared with propofol.The reason could be the slow balance of plasma and effector site concentrations of remimazolam and the combined use of opioids (fentanyl). A comprehensive analysis of three phase 3 clinical trials (Dao et al., 2022) showed that during colonoscopy/bronchoscopy, the need for fentanyl for analgesia was significantly lower in the remimazolam group than in the midazolam group. The results of an animal experiment in cynomolgus monkeys (Kops et al., 2021) showed that the synergistic effect of remimazolam with the opioid remifentanil was significantly higher than that of propofol (94% *versus* 61%). Studies in mice (Bevans et al., 2017) also suggest that remifentanil may enhance the sedative effect of remimazolam, and remimazolam may also enhance the analgesic effect of remifentanil. Therefore, the synergistic sedation effect of fentanyl on remimazolam is stronger than that of propofol at the same dose, which may be more evident in bronchoscopies with deep sedation (our study) and in general anesthesia with endotracheal intubation (Doi et al., 2020). However, in enteroscopy with moderate sedation requirements (MOAA/S ≤ 3), the synergistic effects are smaller because of the low opioid dose and short time. In clinical practice, we can appropriately reduce the dose of remimazolam and improve tolerability by predicting the synergistic effects of different depths of anesthesia. In addition, the use of flumazenil, a remimazolam antagonist, may offer the possibility of even surpassing the recovery speed of propofol.

The incidence of AEs in the remimazolam *versus* propofol groups (74.8% vs. 77.4%,*p* = 0.59) was not statistically significant, the type of AEs was similar, and no SAEs occurred. Except for 1 AE (0.6%) in the experimental group, which was rated as grade 3 (and judged as adverse reactions), the severity of AEs in both the experimental and control groups was 1–2, none of which exceeded 3. Hypotension and injection pain were the most significant differences between the remimazolam group and the propofol group, in which the incidence of hypotension (14.2% vs. 31.6%, *p* < 0.001), hypotension requiring treatment (1.9% vs. 7.7%, *p* = 0.017), and injection pain (0.6% vs. 16.8%, *p* < 0.001) was significantly lower. Hypotension, bradycardia, respiratory depression, and injection pain are common AEs in clinical propofol infusion (Leslie et al., 1995; Marik, 2004; Sim et al., 2009; Desousa, 2016; Dinis-Oliveira,2018). Hypotension and bradycardia may be attributed to myocardial systolic inhibition (Kanaya et al., 2005) and vasodilatory effects (Nagakawa et al., 2003), especially in the elderly or patients with cardiovascular disease (Lee and Shirley, 2021). Injection pain was caused by stimulation of the venous endothelium by the external aqueous phase of propofol emulsion, which can be alleviated by lidocaine and ketamine in clinical practice (Sim et al., 2009; Desousa, 2016).

The incidence of hypotension, injection pain, and respiratory depression were lower with remimazolam than in the propofol group. This has been reported in both surgical procedures with general anesthesia (Doi et al., 2020) and colonoscopies (Chen et al., 2020) in phase III clinical trials. Its excellent safety has also been demonstrated in high-risk surgical patients (ASA class III) (Doi et al., 2020), with no dose-dependent effects on blood pressure reduction. Therefore, the use of remimazolam in bronchoscopy is safe, and the incidence of hypotension and injection pain is significantly lower.

Our results showed no difference in rates of respiratory depression (5.8% vs. 5.2%) and decreased respiratory rate (5.2% vs. 7.1%) between remimazolam and propofol. However, a study by Chen et al. (2020) in colonoscopy showed that the incidence of respiratory depression and decreased respiratory rate was significantly lower with remimazolam compared with propofol. These advantages may have been masked by mechanical ventilation with LMA inserted in our study.

The limitations of our study are as follows: 1) Because bronchoscopy was performed in the outpatient clinic, we administered a fixed dose instead of titration to allow for faster conversion. 2) The safety and efficacy of remimazolam besylate in the high-risk population needs further investigation because the majority of subjects in our study were ASAI-II (99%); 3) The combined use of fentanyl could be a confounding factor because of the different synergistic effects of remimazolam and propofol with opioids (Kops et al., 2021).

In conclusion, the data obtained in this study indicate that 0.2 mg/kg remimazolam besylate for injection is effective and safe for sedation during bronchoscopy. It showed better performance on injection pain and circulatory system effects than propofol-MCT/LCT, but required a slightly longer time for LOC and recovery.

## Data Availability

The original contributions presented in the study are included in the article/Supplementary Material, further inquiries can be directed to the corresponding author.
